# Tuning of Thioredoxin Redox Properties by Intramolecular Hydrogen Bonds

**DOI:** 10.1371/journal.pone.0069411

**Published:** 2013-07-23

**Authors:** Åsmund Kjendseth Røhr, Marta Hammerstad, K. Kristoffer Andersson

**Affiliations:** Department of Biosciences, University of Oslo, Oslo, Norway; Wake Forest University, United States of America

## Abstract

Thioredoxin-like proteins contain a characteristic C-x-x-C active site motif and are involved in a large number of biological processes ranging from electron transfer, cellular redox level maintenance, and regulation of cellular processes. The mechanism for deprotonation of the buried C-terminal active site cysteine in thioredoxin, necessary for dissociation of the mixed-disulfide intermediate that occurs under thiol/disulfide mediated electron transfer, is not well understood for all thioredoxin superfamily members. Here we have characterized a 8.7 kD thioredoxin (BC3987) from *Bacillus cereus* that unlike the typical thioredoxin appears to use the conserved Thr8 side chain near the unusual C-P-P-C active site to increase enzymatic activity by forming a hydrogen bond to the buried cysteine. Our hypothesis is based on biochemical assays and thiolate pK_a_ titrations where the wild type and T8A mutant are compared, phylogenetic analysis of related thioredoxins, and QM/MM calculations with the BC3987 crystal structure as a precursor for modeling of reduced active sites. We suggest that our model applies to other thioredoxin subclasses with similar active site arrangements.

## Introduction

Thioredoxin (Trx) and glutaredoxin (Grx) are small ubiquitous proteins acting as cysteine disulfide oxidoreductases in the cell and belong to the thioredoxin superfamily [1]–[3]. Within this family several classes of proteins have been characterized [4]. They are involved in enzymatic reactions as hydrogen donors [5], [6], in redox signaling [7], protein folding [8], and in the defence against oxidative stress [9]. In general, the Trx-like proteins are reduced by NAD(P)H dependent thioredoxin reductases (TrxR) and they usually have a C-G-P-C active site motif. Grx-like proteins typically contain a C-P-Y-C motif and are reduced by the tripeptide glutathione formed by NADPH utilizing glutathione reductases.

The fundamental mechanism for electron transfer from Trx to its substrate was proposed by Kallis and Holmgren in 1980 [10]. As a result of the lowered pK_a_ value they observed for the N-terminal cysteine thiol (−SH) in the *Escherichia coli* Trx C-G-P-C motif, it was suggested that this thiolate (−S^−^) could perform the initial nucleophilic attack on the substrate disulfide bond. To make the second nucleophilic attack, which dissociates the mixed disulfide intermediate, it was stated that a deprotonation of the buried C-terminal cysteine is necessary. However, the details of this reaction were not discussed. In the following years a number of studies have been focused on the active site thiols and the mechanism behind the proton abstraction from the C-terminal cysteine. It has been suggested that the helix macrodipole [11] or microdipoles [12] are major determinants of Trx cysteine pK_a_ values, however, these explanations have been challenged in later studies [13]. The largest contributions to shifts in pK_a_ values are believed to be intra-protein charge-charge interactions [14], desolvation effects [15], and hydrogen bonding to the deprotonated thiol [16]. A recent study points at the latter effect as the most important [17]. The thiolate form of the N-terminal nucleophilic cysteine positioned at the end of an α-helix, as in Trx, is suggested to be stabilized by intra-protein hydrogen bonding to protein backbone amide protons resulting in a lowered pK_a_ value [18]. Most studies indicate that the buried C-terminal cysteine pK_a_ value is around or higher than ∼8 [10], [19]–[21], thus an explanation for the proton abstraction for this residue is required. A mechanism involving the highly conserved Asp26 and a water molecule functioning as an acid/base catalyst for the buried cysteine has been proposed for *E. coli* and *Acetobacter aceti* Trx [22], [23], however, this mechanism is still debated [24], [25].

Interestingly, other sub-classes of the Trx superfamily with Trx functionality do not have conserved acidic amino acids analogous to the *E. coli* Trx Asp26. Examples are NrdH-redoxins that reduce the active site of the catalytic subunit NrdE of the bacterial class Ib ribonucleotide reductase (RNR) [26]–[28] and *Clostridium pasteurianum* Cp9-type thioredoxins that reduce peroxiredoxins (Prx) [29].

The small thioredoxin BC3987 from *Bacillus cereus* having a significant amino acid sequence similarity with both NrdH and Cp9-redoxins can reduce class Ib RNR in both *B. cereus* and *B. anthracis* [30]. However, the most efficient and likely *in vivo* class Ib RNR reductant in those species is TrxA [31].

Here, we discuss how intramolecular hydrogen bonds can affect deprotonation of the active site cysteines. Substituting the conserved Thr8 that is close to the active site with Ala has altered the hydrogen bond network and the effect of this has been characterized. We have also performed a phylogenetic analysis comparing the amino acid sequence of BC3987 to homologs collected from a range of bacterial species to elucidate which amino acid(s) that contribute to the perturbation of the C-terminal buried cysteine pK_a_ value. The crystal structure of oxidized BC3987 was solved and we have related the results from the phylogenetic analysis to structural features, enabling us to map parts of the protein involved in hydrogen bonds near the active site. Finally, because no structure of reduced BC3987 was obtained, we have modeled the reduced active site using quantum mechanical/molecular mechanical (QM/MM) calculations and rationalized the experimentally observed and unusually low pK_a_ value of the C-terminal buried cysteine through hydrogen bonding to the conserved Thr8 residue. Based on these results, we suggest a general reaction mechanism for the BC3987-like category of thioredoxins.

## Experimental

### Cloning, Mutagenesis, Expression, and Purification of BC3987

Genomic DNA was isolated from *Bacillus cereus* ATCC 14579 using the DNEasy kit from Qiagen. The coding sequence of BC3987 including restriction sites for *XbaI* and *HindIII* was amplified by PCR using the forward primer 5′-CCCTCTAGAAATAATTTTGTTTAACTTTAAGAAGGAGATATACATATGAAAAAAATTGAGGTTTAT-3 and backward primer 5′-AGGAAGCTTAAAAGTTATTCTATATTGAGTAGTTG-3′. The gene was cloned into the pET-22b plasmid (Novagen) and transformed into competent BL21 (DE3) Gold cells (Stratagene). The T8A mutation of BC3987 was generated with the primer 5′-GAAAAAAATTGAGGTTTATGCACAACCCGATTGTCCGCC-3′ using the QuikChange Site-Directed Mutagenesis Kit from Stratagene. A 5 mL overnight culture of BC3987 expressing cells was diluted 1∶200 in 1 litre Terrific Broth medium containing 100 µg/mL ampenicillin and grown until O.D. _600 nm = _0.7–0.8 at 37°C. The cultures were cooled on ice until the temperature reached 15°C and then induced by adding IPTG to a final concentration of 1 mM and the left for 15–16 hours at 20°C in a shaker before harvesting.

Approximately 30 grams of bacteria containing BC3987 was lysed utilizing an X-press [32] and dissolved in 100 mL 100 mM Tris/HCl pH 7.5 containing 10 mM EDTA before centrifugation. DNA was precipitated by adding streptomycin sulfate to a final concentration of 2.5% (w/v). BC3987 was precipitated with 60% ammonium sulfate (0.43 mg/mL). Precipitated protein was dissolved in 50 mM Tris/HCl pH 7.5 and desalted using a HiTrap Desalting column (GE Healthcare). The desalted protein solution was applied to a 1 mL Resource Q anion exchange column (GE Healtcare) and the BC3987 was eluted using a 20 mL 0–400 mM KCl, 50 mM Tris/HCl pH 7.5, gradient. As a final polishing step the protein was purified using a Superdex 200 column (GE Healtcare).

### Protein Analysis and Concentration Determination

The homogeneity of the proteins was analyzed using a Superdex 200 gel filtration column and by SDS gel electrophoresis utilizing the PhastSystem (GE Healtcare) equipped with an 8–25% gradient gel. Protein purity was estimated to >95% by visual inspection of gels. The extinction coefficient of BC3987 was determined using the Edelhoch method [33], yielding a value of εBC3987, 280 nm = 8200 M^−1^cm^−1^.

### Insulin Reduction Assay

The thioredoxin catalyzed reduction of insulin by dithiothreitol (DTT) probes the efficiency of Trx - insulin reduction mechanism. First, both proteins were incubated with 10 mM DTT for 30 minutes and then passed through a HiTrap Desalting column equilibrated with 100 mM potassium phosphate buffer pH 6.5, 2 mM EDTA. The experiments was carried out in 96-well microtiter plates containing 160 µM insulin, 100 mM potassium phosphate buffer pH 6.5, 2 mM EDTA, 1 mM DTT, and 10 µM wt or T8A BC3987 [34]. A Tecan Sunrise plate reader was used to monitor light scattering at 580 nm turbid solution of reduced insulin.

### Estimation of Active Site Cysteine pK_a_ Values

**Figure 1:**
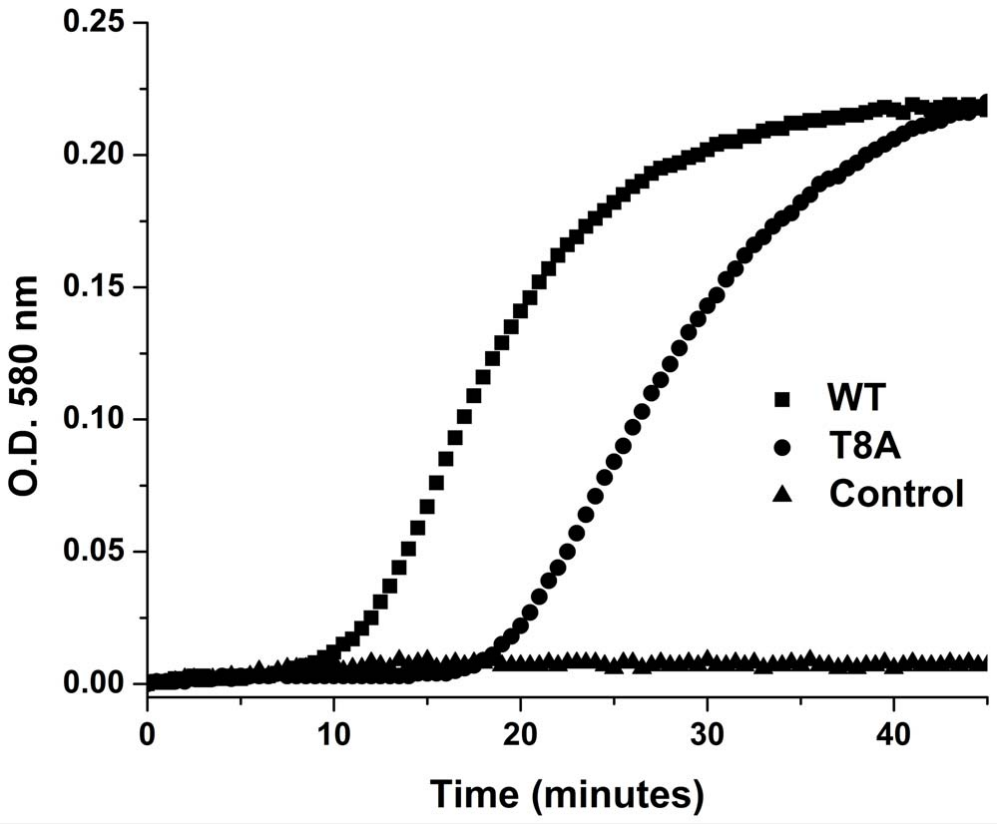
Estimation of active site cysteines pK_a_ values in wild type and mutant BC3987. The pK_a_ values of the active site cysteines in BC3987 were estimated by pH titration resulting in pK_a_ values 5.1 and 7.2 for the wild type and 7.2 for the T8A mutant. A cuvette containing 25 µM reduced protein in 1 mM polybuffer pH 9.3 was added 2 uL aliquots of 25 mM HCl and the change in thiolate absorption at 240 nm were followed with a UV-vis spectrometer.

To estimate the pK_a_ values of the active site cysteines the absorbance of the thiolate anion at 240 nm was followed during a pH titration [35]–[37]. To prepare the reduced wt and T8A mutant BC3987 protein and their corresponding oxidized references, the proteins were incubated with 200 mM DTT or 100 mM diamide for 1 hour, respectively. Excess DTT and diamide was removed using a HiTrap Desalting column (GE Healthcare) equilibrated with a polybuffer with pH 9.3 consisting of 1 mM of each sodium phosphate, sodium citrate, sodium borate, and 0.1 mM EDTA. Spectra of 10–25 µM oxidized and reduced BC3987 was recorded with a HP 8452 diode array spectrophotometer between 200 and 600 nm and corrected for the absorbance difference between the two cuvettes, base line drift, and finally the 240 nm absorbance values were normalized against the 280 nm values. The pH was varied from 9.3 to 2.8 by adding 2–4 µl of 25 mM HCl and measured using a PHM240 pH meter (Radiometer Analytical) equipped with a PHC 4000-8 pH electrode. The equation ε_240 nm_ = A_0_+ A_1_/(1+10^x−pKa1^)+A_2_/(1+10^x−pKa2^) was used to estimate the pK_a_ values by fitting the curves in Figure 1.

### Phylogenetic Analysis

The NrdH amino acid sequences collected from different bacterial divisions were obtained from the Integrated Microbial Genomes (IMG) website [38]. All the NrdH-redoxin amino acid sequences contain the C-[IMV]-Q-C motif and are located in a class Ib RNR operon. Sequences homologous to the Cp9-redoxin found in the anaerobe *C. pasteurianum*, often annotated as YruB proteins after its initial discovery [39], encoding proteins in the range of 75–80 amino acids were downloaded from the UniProt Knowledgebase [40]. Multiple sequence alignment was performed by MAFFT [41] before manual editing using GeneDoc 2.7 (Nicholas and Nicholas, NRBSC). Columns containing gaps and amino acids in the ranges 1–6 and 80–87 in the alignment (Figure S1) were excluded from the phylogenetic analysis. The program Treefinder [42] was used to estimate the substitution model and reconstruct a bootstrapped phylogenetic tree with 1000 replicates at a 50% consensus level utilizing the Maximum Likelihood (ML) algorithm. A second bootstrapped phylogenetic analysis was performed using the Neighbour Joining algorithm included in ClustalX 2.0 [43] using 1000 replicates.

### Crystallization of BC3987

Plate formed crystals were obtained with the Index screen (Hampton Research, CA, USA) condition 55, at room temperature. BC3987 (18 mg/mL) in 15 mM Tris/HCl pH 7.5 was mixed 1∶1 with reservoir solution (0.1 M HEPES pH 7.5, 50 mM MgCl_2_, and 30% (w/v) polyethylene glycol monomethyl ether 550). Crystals of approximately 400×400×30 µm^3^ appeared after 1–2 days. Crystals of mutant BC3987 were obtained at the same conditions as the wild type. So far, crystals with reduced active sites motif have not been obtained when soaking with reductants as DTT and sodium dithionite.

### X-ray Data Collection

Data were collected at the Swiss-Norwegian Beam Line (SNBL, BM01 A) and ID29 at the European Synchrotron Radiation Facility (ESRF), Grenoble, France at 110 K. The protein crystals were flash frozen after 30 seconds incubation in a cryo solution consisting of PEG 400 and reservoir solution in a 1∶6 ratio. The crystals belong to the monoclinic P2_1_ space group and have two protein molecules in the asymmetric unit. Data were integrated with iMOSFLM [44] before scaling and merging with SCALA [45].

### Structure Determination and Refinement

The program PHASER [45], [46] was used to solve the structure of the wild type protein by molecular replacement using a polylalanine search model generated from the *Corynebacterium ammoniagenes* NrdH protein structure [47], PDB ID 1R7H, which has 32% amino acid sequence identity with BC3987. Two rounds of simulated annealing with CNS [48] were followed by several cycles of model building using COOT [49], ARP/wARP [50], and structure refinement in REFMAC5 [51]. The mutant BC3987 crystal structures were solved using the wild type structure as start model. The figures were prepared with PyMOL (W. L. DeLano (2002) PyMOL, DeLano Scientific, San Carlos, CA). The atomic coordinates and structure factors have been deposited in the Protein Data Bank, Research Collaboratory for Structural Bioinformatics, Rutgers University, New Brunswick, NJ.

### QM/MM Calculations Modeling the Reduced Structure

In order to model the reduced active site of BC3987 two-layer ONIOM [52] calculations were performed. The ONIOM procedure allows part of the protein to be treated by quantum mechanical (QM) methods (the high level layer) while a force field based on classical mechanics (MM) is used to describe the rest of the protein (the low level layer). All calculations were executed through the Bioportal (www.bioportal.uio.no) using Gaussian 03, Revision D.02 [53], at TITAN, the computing facility at the University of Oslo. The crystal structure of BC3987 was used as a starting point for modeling the reduced active site. All water molecules were deleted from the structure and hydrogen atoms were added using *GaussView 4.1* (Gaussian, Inc.). The protonated structure was then solvated by adding a 6 Å layer of TIP3P water molecules using the Amber tools 1.2 package [54] and subjected to geometry optimization. In this model preparation step, the coordinates of protein hydrogen atoms and water molecules were geometry optimized using the Amber force field as implemented in Gaussian 03 while keeping the non-hydrogen protein atoms frozen.

In the resulting structure the dihedral angles of Cys12 and Cys15 defined by the atoms N-C_α_-C_β_-S_γ_ were changed by 9° and 46°, respectively, increasing the distance between the two S_γ_ atoms from 2.14 to 3.63 Å. The geometry optimized version of this altered structure is from now on referred to as **Model I**. The precursor of **Model II** has an additional modification, the dihedral angle N-C_α_-C_β_-O_γ_ of Thr8 was changed from 68.9 to 160°, making it resemble the Thr rotamer observed in *E. coli* NrdH (PDBid:1H75 ) [55] and *C. ammoniagenes* NrdH (PDBid:1R7H) [47].

Both models were geometry optimized at the ONIOM(RB3LYP/6-31+G(d,p):UFF) level using the charge equilibrium (QEq) method [56] to assign atomic partial charges. During these calculations, both Cys12 and Cys15 were in their thiolate forms. The high level layer included Tyr7(C) – Gln9(C_α_), the Cys12 side chain, Pro14(C) - Cys15 C_α_, including Cys15 H_Cα_ and side chain, and the Thr53 side chain. All atoms in the high level layer were allowed to move while the coordinates of protein atoms in the low level layer, except Cys12 H_Cα_, C_α_, C, and O and Cys15 C and O, were kept frozen during geometry optimization. The water molecules within a radius of 15 Å of the Cys12 S_γ_-atom were also allowed to move.

## Results

### Characterization of the C-P-P-C Active Site Cysteine pK_a_ Values

Different subclasses of the thioredoxin superfamily have nucleophilic cysteine pK_a_ values that correspond to their biological function. Thus, this is an important parameter when classifying new member of this superfamily.

When estimating the pK_a_ values of the active site cysteines in wild type BC3987 and the T8A mutant, DTT reduced samples were compared with diamide oxidized references. A plot of the thiolate extinction coefficient values plotted against the measured pH is shown in Figure 1. Interestingly, it was observed that the extinction coefficient difference at 240 nm (Δε_240 nm_) due to the thiolate anion was 7–8000 M^−1^cm^−1^. This was a surprising result as an expected Δε_240 nm_ value of about 3500–4000 M^−1^cm^−1^ is typical for a single thiolate [57]–[59]. One possible explanation for this observation is that both cysteines in the reduced C-P-P-C active site have lowered pK_a_ values. The pH-titration curves for wild type and T8A BC3987 in Figure 1 are similar in the pH interval 6.5–9, indicating that the nucleophilic Cys12 pK_a_ value is not affected by the T8A mutation. However, in the pH range 6.5–3 there are clear differences. First, the T8A mutant is not stable below pH 5.4 and precipitates. Second, the wild type curve has a shoulder between pH 4 and 6.5, indicating a titratable thiolate with a lowered Δε_240 nm_. A lowered Δε_240 nm_ for the buried Cys15 can be rationalized because the absorbance maximum of thiolates depends on the polarity of the solution, shifting to lower wavelengths with decreasing polarity. Following the reasoning above, the wild type BC3987 has two titratable thiolates with pK_a_ values 5.1 and 7.2 while the T8A mutant has one thiolate with a pK_a_ value of 7.2 and a second thiolate that behave different from the wild type.

### BC3987 Wild Type and the T8A Mutant Reduction of Insulin Disulfides

**Figure 2:**
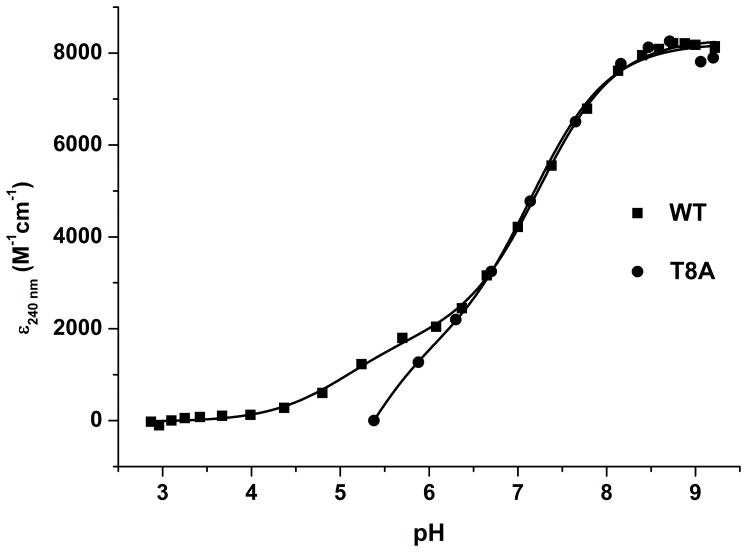
Insulin reduction by wild type and mutant BC3987. In the insulin reduction assay, the rate of insulin disulfide reduction by DTT is enhanced using 10 µM thioredoxin as a catalyst. The wild type BX3987 thioredoxin (▪) is more efficient than the T8A mutant (•). The control experiment without thioredoxin shows that the uncatalyzed reaction with DTT is very slow (▴). The concentration of insulin and DTT used in the assay was 160 µM and 1 mM, respectively. The turbidity of the assay solution was monitored at 580 nm (light scattering).

In the insulin reduction assay, thioredoxin catalyze the reduction of insulin by DTT and the light scattering of reduced insulin is measured. As shown in Figure 2, the control reaction with insulin and DTT is very slow compared to the wild type and T8A mutant BC3987 catalyzed reactions. The efficiency of thioredoxin insulin reduction is measured in this assay, and from the curves in Figure 2 it can be concluded that the wild type BC3987 has a different reactivity towards the substrate compared to the T8A mutant. The lag-time before any precipitated insulin is observed is much longer for the T8A mutant, and the slope of the curve after the lag-period is slightly steeper for the wild type enzyme.

### Phylogenetic Analysis

**Figure 3:**
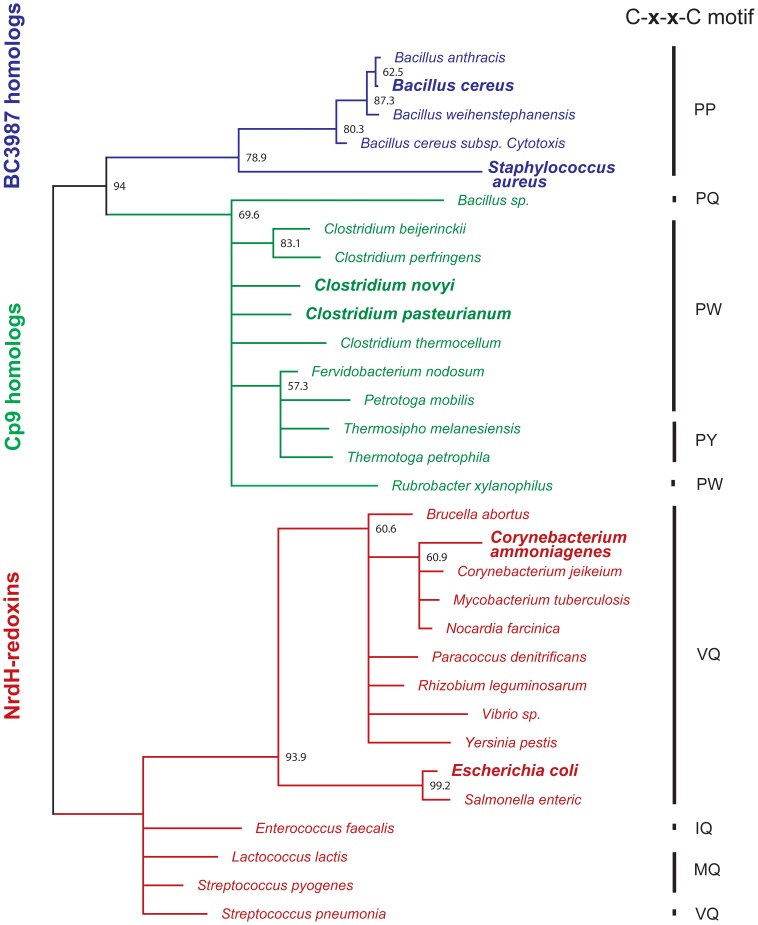
Phylogenetic analysis of thioredoxins similar to BC3987. Bootstrap values obtained at a 50% consensus level using the Maximum Likelihood algorithm with 1000 replicates are shown at the branches of the tree. The evolutionary distances from the node separating the three clusters BC3987 homologs (in blue), Cp9 homologs (in green), and NrdH-redoxins (in red) are 0.47, 0.44, and 0.50 substitutions per site, respectively. Among the BC3987 homologs the active sites always have the -C-P-P-C- motif while the classical glutaredoxin active site motif -C-P-[YW]-C- is prevalent for the Cp9-like proteins. For the NrdH-redoxins, all encoded in a *nrdHIEF* operon, the predominant active site motif is -C-V-Q-C-. Alignment shown in Figure S1 (same color coding).

The BC3987 homologs within the *B. cereus* group have 28–32% sequence identity to NrdH-redoxins and 37% sequence identity to the *Clostridium novyi* Cp9-redoxin homolog. Thus, it was of interest to compare these three groups that all belong to the thioredoxin superfamily. The analysis was performed using the method of Maximum Likelihood with a WAG [60] substitution model, based on the AICc criterion [61], [62] with optimized frequencies, proposed by the Treefinder program. In the phylogenetic tree shown in Figure 3, a node separates the three groups at a 94% consensus level. The tip-to-tip distances between the node and the three groups, *Bacillus cereus* group BC3987 homologs (Blue), Cp9 homologs (Green), and NrdH-redoxins (Red) are 0.47, 0.44, and 0.50 substitutions per site, respectively.

A multiple amino acid sequence alignment of the *B. cereus* group BC3987 homologs, NrdH-redoxins, and Cp9 homologs included in the phylogenetic analysis shows that several residues are highly conserved for all classes in addition to the cysteines in the C-x-x-C motif (Figure S1). Using the amino acid numbering from BC3987 the Pro54, Gly65, and Phe66 are conserved for all the sequences included in the alignment. In addition, a N-terminal [IV]-X-[ILV]-[YF]-[ST] motif ranging from residue 4 to 8 and a [LI] residue at position 71 seems to be conserved within all classes. The active site motif (Figure 2, right side) has a larger variation in the Cp9 homologous proteins (C-[PGI]-[PYWQ]-C) than the NrdH-redoxins (C-[VMI]-Q-C), however, the Grx-like C-P-[YW]-C motif seems to be the most common within the Cp9 group. BC3987 and Cp9 homologous sequences have an insert at position 44 that is a Leu or Met residue that is not present in the NrdH proteins (Figure S1). The other *B. cereus* group BC3987 homologs also have this insert, supporting the co-classification with Cp9. From these results we suggest that the *Bacillus* genus BC3987 homologs, Cp9 homologs, and NrdH-redoxins form three evolutionary separated groups.

However, due to the occurrence of the same amino acid insertion and the higher sequence identity observed for the *Bacillus* genus BC3987 homolog group and the Cp9 homologs one might expect a closer structural, and perhaps functional, relationship for these two groups compared to the NrdH-redoxins.

### Identification of Conserved Amino Acids Preceding the C-x-x-C Site in Thioredoxin-like Proteins

In general the local environment of the active site cysteines is considered to be very important with regard to reactivity and redox potential of Trx. Within the 6 amino acids preceding the C-x-x-C motif there are often highly conserved Asp, Thr, and Ser residues that have been shown to affect the activity of the enzyme or are suggested to do so. These residues are located on a β-strand that folds back on the active site, thus the amino acid side chains in position 2, 4, and 6 can point towards the buried cysteine. All proteins in the IMG database having less than 160 amino acids and containing either x(6)-C-G-P-C (Trxs), x(6)-C-P-[FYW]-C (Grxs), x(6)-C-[VIM]-Q-C (NrdH-redoxins), or x(6)-C-P-P-C motifs (PROSITE syntax used to describe motifs) and being annotated as either Trxs, Grxs, or NrdH-redoxins, have been analyzed with focus on the [DE]-x-[ST]-x(3) motif preceding the active site (Table 1).

**Table 1:** Categorization of active site motifs in thioredoxin-like proteins (% of sequences)^a^_._

	x-x amino acids			
*Active site motif* b	*Thioredoxin*	*Glutaredoxin*	*NrdH*	*Other*
	G-P	P-[FYW]	[VIM]-Q	P-P
x(6)-C-x-x-C	61.1	25.2	8.5	5.2
x(2)-[ST]-x(3)-C-x-x-C	3.0	15.1	8.5	2.7 (2.2 T, 0.5 S)
[DE]-x(5)-C-x-x-C	56.5	0	0	1.3
[DE]x[ST]-x(3)-C-x-x-C	1.5	0	0	0.3

a Analysis of 2627 sequences. All sequences between 60 and 160 amino acids in the Integrated Microbial Genomes database (http://img.jgi.doe.gov) containing a x(6)-C-x-x-C motif were downloaded. Sequences that were not annotated or did not have a Pfam classification as a member of the thioredoxin superfamily were deleted prior to the analysis.

b PROSITE syntax (http://au.expasy.org/tools/scanprosite/scanprosite-doc.html#pattern_syntax) is used to describe the amino acid motifs preceeding the N-terminal active site Cys.

### Quality of the BC3987 Crystal Structure

The BC3987 wild type and T8A mutant structures were refined to 1.4 and 1.18 Å, respectively. In the wild type structure residues 1–76 and 3–75 are observed in chain A and B, respectively. Chain A shows the overall highest quality with residues 1–76 visible in the electron density map, while residues Lys35, Lys36, Phe66, Glu69, and Gln72 in chain B lack density at a few side chain atoms. In the T8A mutant structure, all residues 1–78 are visible in the electron density map of the corresponding high quality chain. In the other chain, residues 3–76 are observed, with lacking density at the side chain atoms of Asn76. Refinement and validation statistics are presented in Table 2.

**Table 2:** Crystal data, data collection, and refinement statistics.

*Crystal data*		
	Wild type	T8A mutant
Space group	P2_1_	
Crystal parameters	a = 24.7, b = 98.9, c = 25.1	a = 24.4, b = 99.1, c = 25.2
	α = 90, β = 91.8, γ = 90	α = 90, β = 91.6, γ = 90
***Data collection***		
X-ray source	ESRF, ID29	ESRF, ID29
Resolution (Å)^a^	33–1.4 (1.48–1.4)	24.8–1.18 (1.25–1.18)
Wavelength (Å)	0.975948	0.976273
Temperature (K)	100	100
Completeness (%)^a^	94.3 (93.8)	84.9 (83.1)
Redundancy (%)^a^	2.6 (2.6)	3.1 (3.0)
I/σ(I)^a^	15.0 (6.1)	13.1 (4.9)
R_sym_ (%)^b^	0.03 (0.11)	0.04 (0.18)
***Refinement statistics***		
R_cryst_ (%)^c^	18.8	14.4
R_free_ (%)^d^	20.5	19.7
Mean overall isotropic B-factor (Å^2^)	19.9	20.8
Ramachandran plot: ration in most favored/other allowed regions (%)		
RMS deviation from standard bond lengths (Å)/angles (°)	0.026/2.44	0.026/2.40
Added waters	98	88
**PDB code**	3ZIJ	3ZIT

a Values for outer shell in parenthesis.

b R_sym_ = Σ|I–〈I〉|/ΣI.

c R_cryst_ = Σ(|F_obs_|–|F_calc_|)/Σ|F_obs._|.

d R_free_ is the R_cryst_ value calculated on the 5% reflections excluded for refinement.

### Overall Structure of BC3987

Like *E. coli* and *C. ammoniagenes* NrdH the BC3987 have a α/β/α Trx fold typical for all members of the Thioredoxin superfamily. The structure has a C_α_ RMSD value of 1.26 Å with the *E. coli* NrdH (PDBid:1H75). Due to the insert of one Leu amino acid at position 44 in the BC3987 protein, the α2 helix is positioned slightly closer to the β-sheet layer compared to *E. coli* NrdH. The amino acid insert not found in NrdH-redoxins (Leu44) points into the cavity on the BC3987 surface that resembles the binding site for TrxR described by Lennon *et al.* [63].

### The C-P-P-C Active Site Motif

**Figure 4:**
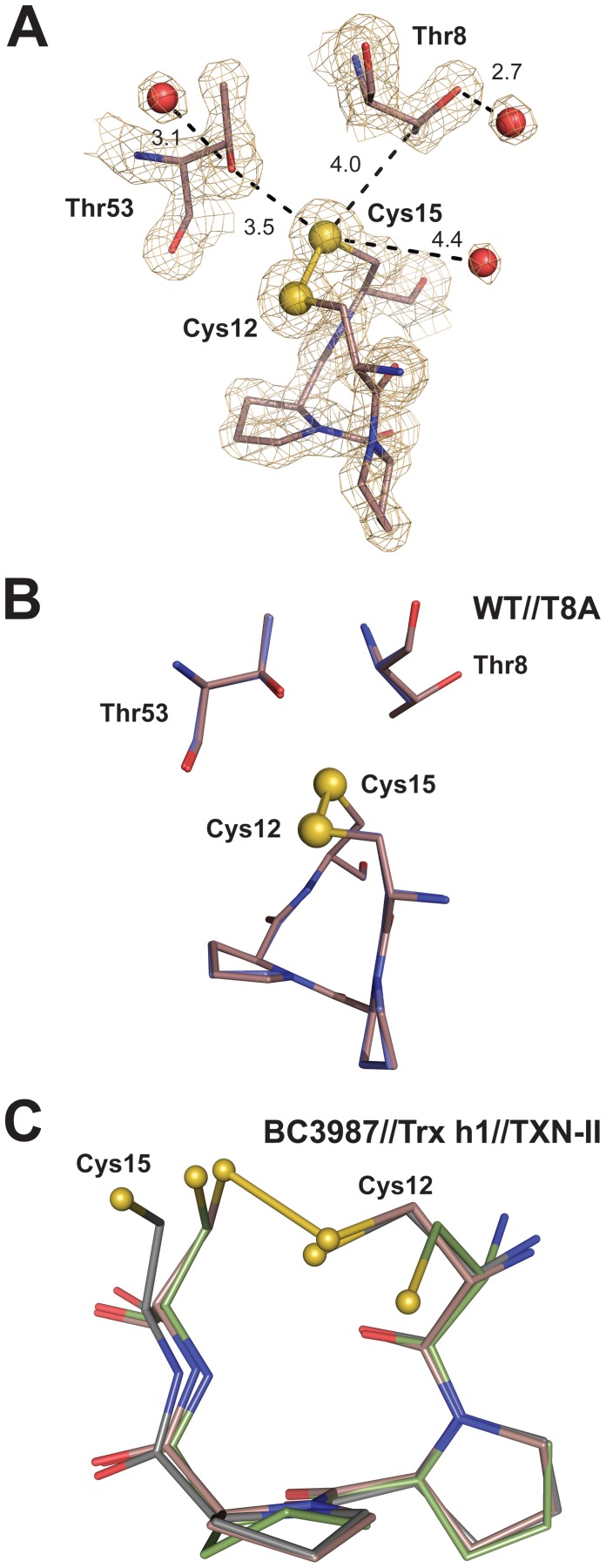
Structure of BC3987 active site. (A) The active site and immediate surroundings in BC3987. Thr8 and Thr53 can possibly form hydrogen bonds to the S_γ_-atom of Cys15 upon reduction of the disulfide bridge. The |2F_o_–F_c_| map is contoured at 1.5 σ. (B) The wild type (in brown) and T8A (in blue) crystal structures have CPPC active sites that superimpose with a RMS value of 0.053 Å. This verifies that the mutation does not disturb the CPPC active site. (C) Comparison of C-P-P-C motifs in oxidized BC3987 (in brown), reduced Tryparedoxin, TXN-II, (in green), and reduced Trx h1 (in grey). From this superimposition, it is suggested that the cysteine side chains and not the backbone undergo the largest structural rearrangement upon reduction of the active site.

The BC3987 protein has a C-P-P-C active site motif that differs from what is typically observed for Trxs and Grxs. Additionally, the residues Thr8 and Thr53 in the vicinity of the disulfide bridge are possible hydrogen bond partners for the buried C-terminal cysteine (Figure 4A). Contrary to what is seen in the NrdH-redoxins from *E. coli* and *C. ammoniagenes* crystal structures the Thr8 residue in BC3987 has a rotamer where the hydroxyl group is pointing away from the buried Cys15 S_γ_-atom. This orientation of Thr8 is stabilized by a hydrogen bond to a water molecule (HOH34). The proline residues apply restrictions on the protein backbone, making a rigid frame for the active site cysteines. Both proline residues in the BC3987 active site have the trans-conformation with ω-angles of 177° and 176° for the Pro13 and Pro14, respectively. These values fall within the ω-angle standard deviation observed for trans-proline residues with the UP-pucker [64], indicating that the motif probably is structurally relaxed. The crystal structure of the T8A mutant resembles the wild type structure and the C-P-P-C motifs in these structures superimpose with a RMSD value of 0.053 (Figure 4B). A superimposition of the oxidized C-P-P-C motif in BC3987 and the corresponding reduced C-P-P-C motifs found in Tryparedoxin-II (TXN-II) from *Crithidia fasciculate* (PDBid:1FG4) and Poplar Trx *h*1(PDBid:1TI3, model 3) is shown in Figure 4C. The RMS values calculated for all C-P-P-C atoms except Cys-C_β_/S_γ_ are 0.32 and 0.22 Å with Tryparedoxin II and Trx *h*1, respectively, indicating that the disulfide redox state does not influence the active site conformation notably.

### QM/MM Modeling of the Reduced Active Site Motif

Based on the results from the Cys pK_a_ titration experiments and the different Thr rotamers observed in the crystal structures of BC3987 and the NrdH-redoxins one might speculate that the conserved Thr8 could contribute to lower the buried cysteine pK_a_ value through hydrogen bonding. In the structure it is apparent that the Gln9 amide proton is within hydrogen bonding distance to the S_γ_-atom of Cys15, however, a Thr8 with a rotamer as observed in *E. coli* and *C. ammoniagenes* NrdH-redoxins could also act as a hydrogen bond donor to this atom. Additonally, it was of interest to examine if the variable Thr53 could form a hydrogen bond to the buried cysteine thiolate when BC3987 was in the reduced form.

**Figure 5:**
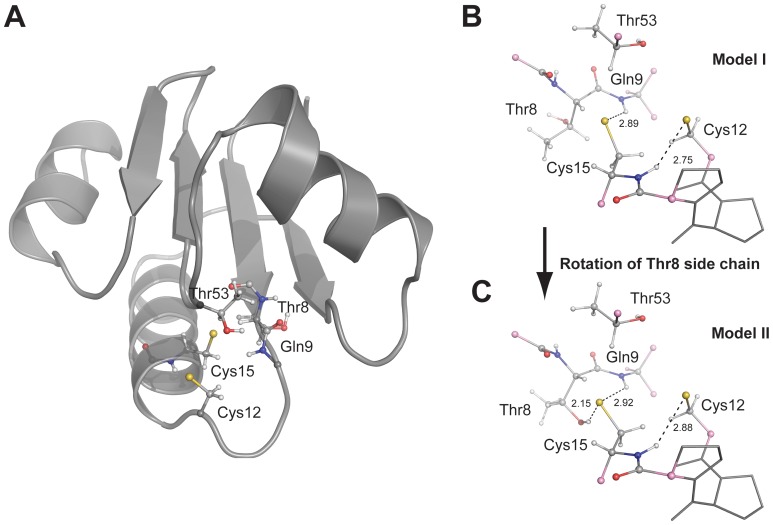
QM/MM models of the BC3987 active site. (A) QM/MM geometry optimized structure of BC3987 showing the quantum mechanical and molecular mechanics regions in ball-and-stick and cartoon representation, respectively. (B) Active site (Model I) where the Thr8 residue has the rotamer observed in the crystal structure. The Cys12 side chain is hydrogen bonded to the Cys15 amide proton. The Gln9 amide proton is a possible hydrogen bond donor to the buried Cys15 thiolate. (C) In this structure (Model II) the Thr8 side chain was rotated to resemble the rotamer observed in *E. coli* and *C. ammoniagenes* NrdH-redoxins prior to geometry optimization. The Thr8 hydroxyl group and Gln9 amide proton appear to form hydrogen bonds to the buried Cys15 side chain while the Cys12 side chain is hydrogen bonded to the Cys15 amide proton. The link atoms connecting the MM and QM layer in B and C are colored pink.

To be able to investigate the effects of Thr8 and Thr53, a QM/MM modeling approach including the whole protein was chosen. The largest part of the protein (the low layer, cartoon representation in Figure 5A) was modeled by a classical force field, while hybrid density functional theory was used to describe the active site and its immediate surroundings (the high layer, ball-and-stick representation in Figure 5A). Geometrical restraints were applied on the active site to mimic the limited conformational difference between red-ox states observed in Tryparedoxin-II and Poplar Trx *h*1 (Figure 4C). From the two models of the reduced active site, Model I with Thr8 having the rotamer observed in the BC3987 crystal structure (Figure 5B) and Model II where the conformation of Thr8 resemble the corresponding Thr residue in NrdH-redoxins (Figure 5C), there are several differences. Hydrogen bonding distances within the active site in Model I and II have been summarized in Table 3. Additionally, the pK_a_ values of the cysteines in both geometry optimized structures were estimated using the program PROPKA 2.0 [17], and the perturbations caused by the individual hydrogen bonds are also shown in Table 3.

**Table 3:** Hydrogen bond distances^a^ to active site thiolates and their estimated perturbation ΔpK_a_ b on the pK_a_ values.

*Donor atom*		Thr53-O_γ_	Thr8- O_γ_	Gln9-N_amide_	Cys15-N_amide_	Estimated pK_a_
*Model I*	Cys12	4.38 Å	–	–	3.70 Å	**7.1**
		−0.19^b^	–	–	−1.74^b^	
	Cys15	4.20 Å	–	3.68 Å	–	**8.0**
		−0.49^b^	–	−1.07^b^	–	
*Model II*	Cys12	4.41 Å	–	5.26 Å	3.80 Å	**7.1**
		−0.14^b^	–	−0.08^b^	−1.70^b^	
	Cys15	–	3.09 Å	3.71 Å	–	**6.4**
		–	−1.60^b^	−1.66^b^	–	
*Experiment*	Cys12					**7.2**
	Cys15					**5.1**

a Hydrogen bonding distances obtained from the geometry optimized structures Model I and Model II.

b The perturbation of the thiolate pK_a_ values due to hydrogen bonding were calculated using the program PROPKA 2.0 (http://propka.ki.ku.dk/).

In both models the nucleophilic Cys12 side chain is hydrogen bonded to the Cys15 amide proton, rationalizing the perturbed pK_a_ value of this thiol. It is also clear that the amide protein of Gln9 can form a hydrogen bond to the buried Cys15 thiolate in both Model I and II. A limited perturbation of the variable Thr53 residue on the Cys12 pK_a_ value is predicted, the effect being similar in magnitude for both models. Indeed, an additional hydrogen bond from Thr8 to the buried cysteine thiolate can be observed in Model II (Figure 5C), resulting in calculated pKa values of 7.1 and 6.5 for Cys12 and Cys15, respectively. The corresponding values for Model I are 7.1 and 8.0, indicating that Model II is most compatible with experimental observations and supporting the hypothesis that Thr8 is involved in the stabilization of the Cys15 thiolate. With respect to computed energies, the high layer B3LYP energy is 8.7 kcal/mol lower for Model II compared to Model I, however, the total ONIOM energy for Model II is 11.8 kcal/mol higher than for Model I. This indicates that effects from the protein low layer disfavor Model II compared to Model I, while the active site electronic structure in Model II is stabilized by the hydrogen bond between Thr8 and Cys15.

## Discussion

The thioredoxin BC3987 found in *B. cereus* ATCC 14579 is conserved among all the members of the *B. cereus* group, including *B. anthracis, B. mycoides, B. pseudomycoides, B. thuringiensis* and* B. weihenstephanensis*. It has been shown that BC3987 can function as an electron donor to class Ib RNR [30]. However, subsequent studies using the corresponding enzymes from *B. anthracis* showed that the BC3987 homolog is 6–7 times less effective than Trx1 in reducing RNR and that Trx1 is 60 fold abundant compared to the BC3987 homolog [31]. Thus, it is most likely that Trx1 is the *in vivo* electron donor to class Ib RNR in both *B. anthracis* and *B. cereus.*

**Figure 6:**
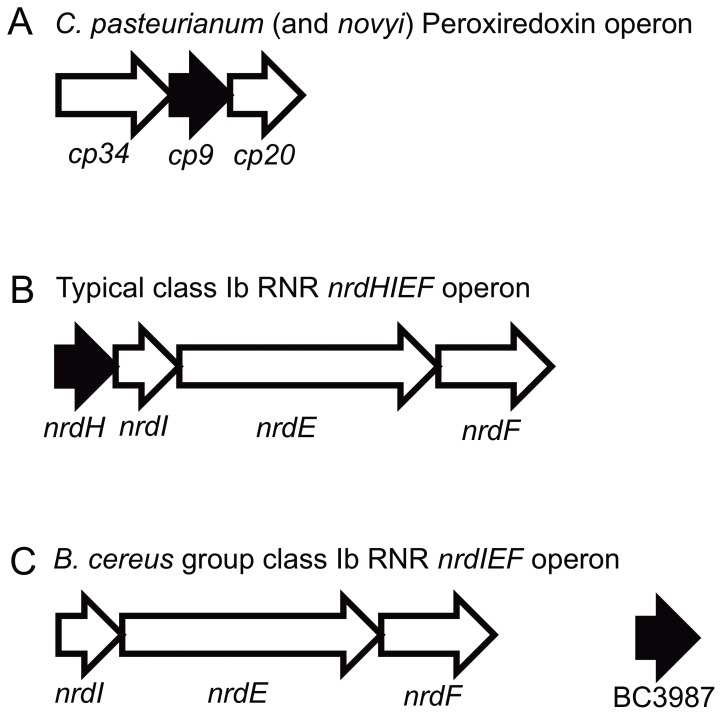
Overview of operon organization of Cp9-redoxins, NrdH-redoxins, and BC3987. (A) The gene encoding the Cp9-redoxin is located between a thioredoxin reductase (cp34) and a peroxiredoxin (cp20). (B) The classical class Ib RNR operon where the NrdH-redoxin is found in front of the genes encoding the flavodoxin-like protein NrdI, the catalytic subunit NrdE and the radical/metal cofactor containing NrdF protein. (C) The organization of class Ib RNR genes in the *Bacillus cereus* group where the putative NrdH-redoxin (BC3987) is located elsewhere in the genome.

The protein showing the highest amino acid identity (37%) to BC3987 does not belong to a member of the *Bacillus* genus, but is a small Trx (NT01CX_2375) found in the anaerobe *C. novyi*. This protein has 58% sequence identity with the *C. pasteurianum* thioredoxin Cp9 that is encoded in an operon between a NADH dependent TrxR and a Prx (Figure 6A), functioning as an electron transporter between these two proteins [29]. The NT01CX_2375 gene is found in an identical operon structure as Cp9 with flanking TrxR and Prx. These three proteins have been found to be present at relatively high levels in *C. novyi* spores [65]. *B. cereus* ATCC 14579 has a gene (BC2114) encoding a protein that has 54% sequence identity to the *C. novyi* Prx, however, this gene is not colocated with any TrxR or Trx genes. Thus, one possible substrate for BC3987 is the putative Prx BC2114. Aside, it should be mentioned that several non-bacterial Prxs with ∼50% sequence identity to BC2114 have been characterized, and that some of them are reduced by redoxins with C-P-P-C active site motifs as found in BC3987 [66], [67].

In order to relate BC3987 to the Cp9-redoxins found in *C. pasteurianum*/*novyi* and NrdH-redoxins, a phylogenetic analysis of a collection of sequences representing these proteins were carried out. The tree shown in Figure 2 indicates that the BC3987 homologs found among the members in the *Bacillus cereus* group form a separate cluster from the NrdH-redoxins and the proteins homologues to the C. *pasteurianum*/*novyi* Cp9 thioredoxin. A slightly shorter evolutionary distance is observed between the clusters represented by BC3987 and the C. *pasteurianum*/*novyi* Cp9 compared to that formed by NrdH-redoxins. The amino acid deletion at position 44 in the NrdH-redoxins was not taken into account while performing the phylogenetic analysis, thus, this deletion increase the evolutionary distance of the NrdH-redoxins to the other two groups further. The role of BC3987 as a part of Prx-mediated defense against reactive oxygen species in *B. cereus* is currently investigated in our laboratory.

The only two cysteine residues in the protein are found in the active site, and the observation that the change in the molar extinction coefficient Δε_240 nm_ was about twice of the expected value for one thiol/thiolate pair indicate that both active site cysteines have lowered pK_a_ values. Similar observations have been made for the *B. subtilis* thiol-disulfide oxidoreductases ResA and StoA where a water molecule bound to a Glu residue can hydrogen bond to the C-terminal buried cysteine and lower its pK_a_ value [68], [69]. The mechanism for lowered thiol pK_a_ values in C-x-x-C active sites involved in redox chemistry is discussed below.

In Figure 3 it is shown that the C-x-x-C motifs among established and putative Trxs have substantial variation. The typical Trx active site motif is C-G-P-C as seen in Table 1 where a large number of thioredoxin superfamily members have been analyzed and compared, also including the six amino acids preceding the C-x-x-C motif.

About 92% of the typical Trxs included in the survey have an acidic Asp or Glu residue in the sixth position preceding the C-G-P-C motif, and it has been proposed that this residue acts as a general acid/base catalyst for proton transfer during reduction and oxidation of the disulfide bridge [22]. For *E. coli* Trx, this residue, which is located 5.6 Å away from the buried C-terminal active site cysteine, has been demonstrated to be essential for catalytic efficiency [21]. It has also been suggested that a Ser or Thr residue four residues in front of the C-x-x-C motif, [ST]-x(3)-C-x(2)-C, influence Trx activity by interacting directly with the previously discussed Asp residue [70] or the buried Cys residue [71]. Yet, less than 5% of the C-G-P-C type Trxs included in our survey has a Ser or Thr residue in this position (Table 1), indicating that such an arrangement is not a general feature for this type of Trxs. None of the Grxs or NrdH-redoxins have an Asp or Glu in the sixth position. However, the [ST]-x(3)-C-x(2)-C motif is observed in 60 and 99.5% of Grxs and NrdH-redoxins (Table 1), respectively. As we have shown in this work, mutation of the Thr8 residue in the forth position preceding the C-P-P-C motif decrease the catalytic efficiency of the enzyme (Figure 2).

In the structures of the oxidized NrdH-redoxins from *E. coli* and *C. ammoniagenes* the hydroxyl O- atom of the Thr residue in T-x(3)-C-V-Q-C motif is oriented towards the buried Cys-S_γ_ with distances of 3.4 and 3.8 Å, both being potential hydrogen bonding partners with the cysteine thiol/thiolate when the active sites are in their reduced states.

In BC3987, which has a ^6^V-Y-T-Q-P-D-C-P-P-C^15^ motif, Thr8 is a potential hydrogen bond donor to Cys15-S_γ_. However, in the BC3987 crystal structure the side chain of this residue is hydrogen bonded to a water molecule resulting in a rotamer where the Thr hydroxyl group points away from the buried Cys15 as shown in Figure 3A, different from what observed in the *E. coli* and *C. ammoniagenes* NrdH-redoxins. Considering the similarity of the Thr8 environment with those of Thr7 in the *E. coli* and *C. ammoniagenes* NrdH-redoxins, a low energy barrier between the two rotamers seems likely. In addition, the Thr53 residue, not conserved within the NrdH and Cp9-redoxin families, was found in the vicinity of the active site. Thus, there are two potential hydrogen bond donors to the Cys15 S_γ_-atom in addition to the Gln9 amide protein.

**Figure 7:**
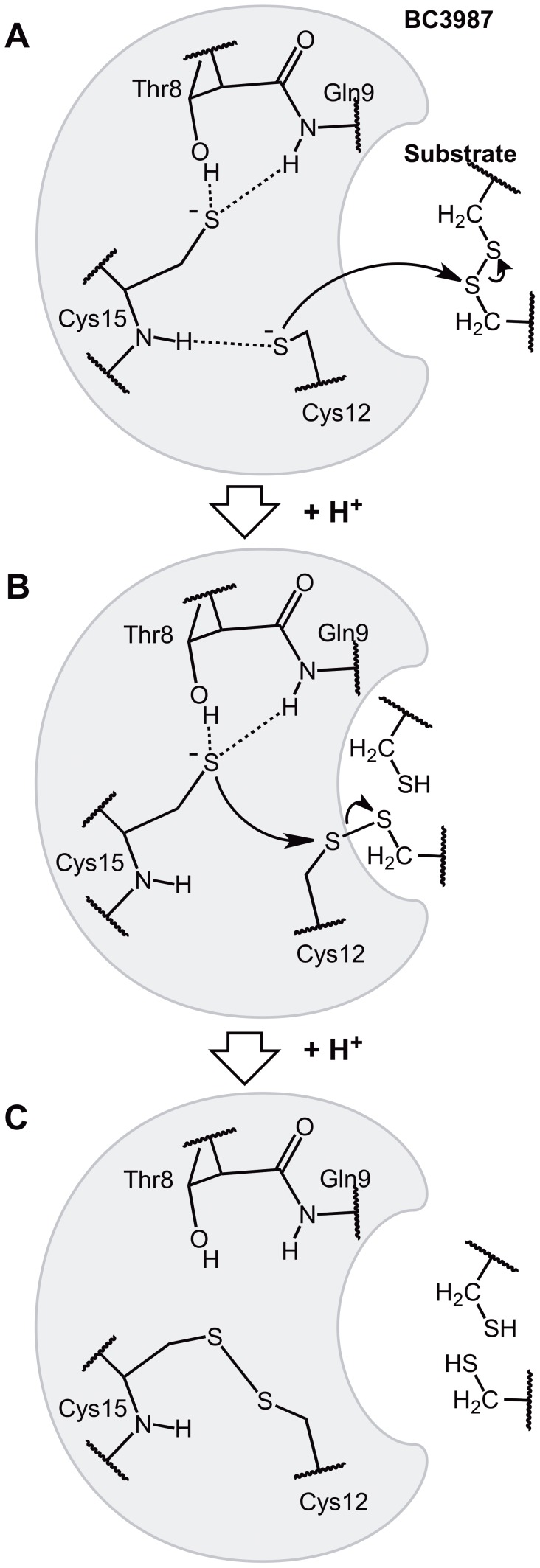
A possible substrate reduction reaction mechanism for BC3987 based on Model II involving two active site thiolates. (A) The reaction is initiated by a nucleophilic attack on the substrate disulfide bridge by the Cys12 thiolate and a mixed disulfide intermediate is formed. (B) The buried Cys15 S_γ_-atom that is stabilized in its thiolate form by hydrogen bonding toThr8 and Gln9 perform a second nucleophilic attack on the Cys12 S_γ_-atom. (C) The reduced substrate is released and the two active site cysteines in BC3987 form a disulfide bridge.

The modeling studies of the reduced active site provided information on its possible hydrogen bond arrangements. Two models were examined using QM/MM calculations, one where Thr8 had the rotamer present in the BC3987 crystal structure (Model I, Figure 5B) and one where the Thr8 side chain had been rotated to resemble the conformation found in *E. coli* and *C. ammoniagenes* NrdH-redoxins (Model II, Figure 5C). The experimentally observed lowered pKa values for the buried cysteine thiol, can be explained through hydrogen bonding from the Gln9 amide protein and the Thr8 side chain. Showing that the *E. coli* and *C. ammoniagenes* NrdH-redoxin Thr8 rotamer is capable to form a hydrogen bond to the buried cysteine has important implications for all Trxs having the [ST]-x(3)-C-x-x-C motif. Such an interaction can result in a stabilization of the buried cysteine thiolate in the active site at physiological pH values, making a simple reaction mechanism like the one presented in Figure 7 plausible. In this mechanism, the initial nucleophilic attack is carried out by the solvent exposed Cys12 that is stabilized in its deprotonated thiolate form due to hydrogen bonding to the Cys15 amide protein (Figure 7A). This primary activation of the nucleophilic cysteine could be general to all Trxs. To break the resulting mixed disulfide intermediate the buried Cys15 needs to be deprotonated to perform the second nucleophilic attack. We suggest that Cys15 is already in its deprotonated form, stabilized by hydrogen bonding to Thr8 and Gln9 (Figure 7B). Thus, this mechanism offers an elegant explanation of the deprotonation of the buried cysteine in Trxs that lack an analog to the Asp26 residue in *E. coli* Trx.

In summary it has been shown that BC3987 appears to be a closer relative to Trxs that reduce peroxiredoxins than NrdH-redoxins that provide electrons to the catalytic subunit of class Ib ribonucleotide reductases. The observation that the reduced form of BC3987 seems to possess two cysteine residues in the active site with lowered pK_a_ values have been explained through intra-protein hydrogen bonding patterns. Disturbing the hydrogen bonding networks by removing a highly conserved Thr residue near the buried Cys resulted in decreased efficiency for the BC3987 thioredoxin.

## Supporting Information

Figure S1 Multiple alignment of amino acid sequences of NrdH-redoxins (in red), Cp9/NT01CX_2375 homologs (in green), and BC3987 homologs (in blue). The columns containing the conserved Thr/Ser residues in position 8, and the gaps in the NrdH-redoxin sequences in position 44, are marked with arrows and with orange background.
(PDF)
