# Correction: Tuning of Thioredoxin Redox Properties by Intramolecular Hydrogen Bonds

**DOI:** 10.1371/annotation/2e2b21bd-bbf7-4a4b-a9cf-194b693e02bf

**Published:** 2013-08-06

**Authors:** Åsmund Kjendseth Røhr, Marta Hammerstad, K. Kristoffer Andersson

Figures 1 and 2 were accidentally switched. The image appearing as Figure 1 belongs with the title and legend for Figure 2, and the image appearing as Figure 2 belongs with the title and legend for Figure 1. The titles and legends are in correct order. 

