# Supplementary material for: Tuning of Thioredoxin Redox Properties by Intramolecular Hydrogen Bonds
Source: PLoS One. 2013 Jul 23;8(7):e69411. doi: 10.1371/journal.pone.0069411 (PMC3720550; doi:10.1371/journal.pone.0069411)
Supplement: Figure S1 — Multiple alignment of amino acid sequences of NrdH-redoxins (in red), Cp9/NT01CX_2375 homologs (in green), and BC3987 homologs (in blue). The columns containing the conserved Thr/Ser residues in position 8, and the gaps in the NrdH-redoxin sequences in position 44, are marked with arrows and with orange background. (PDF) [file pone.0069411.s001.pdf]

Figure S1

Tuning of Thioredoxin Redox Properties by Intramolecular Hydrogen Bonds

Åsmund Kjendseth Røhr, Marta Hammerstad, and K. Kristoffer Andersson

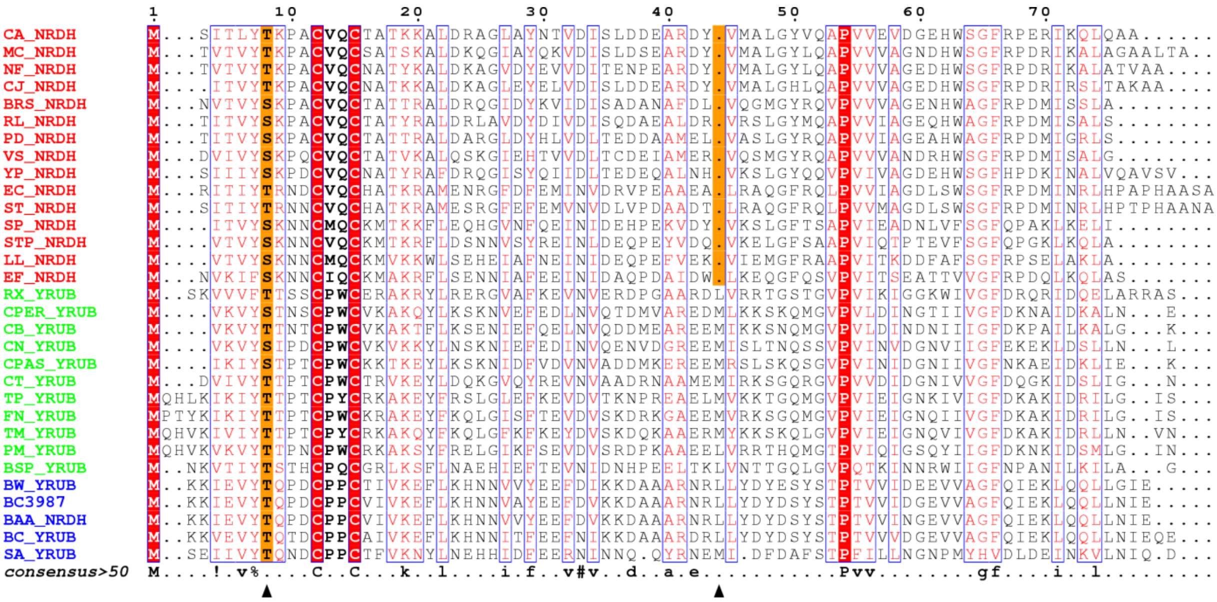

Figure S1. Multiple alignment of amino acid sequences of NrdH-redoxins (in red), Cp9/ NT01CX_2375 homologs (in green), and BC3987 homologs (in blue). The columns containing the conserved Thr/ Ser residues in position 8, and the gaps in the NrdH-redoxin sequences in position 44, are marked with arrows and with orange background.

The corresponding species and accession numbers of the sequence names are as follows: CA_NRDH; *Corynebacterium ammoniagenes* (O69271), MC_NRDH; *Mycobacterium tuberculosis* (NP_217569), NF_NRDH*;* *Nocardia farcinica* (YP_120522), CJ_NRDH; *Corynebacterium jeikeium* (YP_250251), BRS_NRDH; *Brucella abortus* (YP_001932759), RL_NRDH; *Rhizobium leguminosarum* (YP_769836), PD_NRDH; *Paracoccus denitrificans* (YP_001607868), VS_NRDH; *Vibrio sp.* (ZP_01066345), YP_NRDH; *Yersinia pestis* (YP_001873486), EC_NRDH; *Escherichia coli* (ZP_03083809), ST_NRDH; *Salmonella enteric* (YP_002115761), SP_NRDH; *Streptococcus pyogenes* (NP_269480), STP_NRDH; *Streptococcus pneumonia* (YP_002037786), LL_NRDH; *Lactococcus lactis* (YP_001032827), EF_NRDH; *Enterococcus faecalis* (NP_814257), RX_YRUB; *Rubrobacter xylanophilus* (YP_644383), CPER_YRUB; *Clostridium perfringens* (ZP_02953769), CB_YRUB; *Clostridium beijerinckii* (YP_001307271), CN_YRUB(NT01CX_2375); *Clostridium novyi* (YP_878448), CPAS_YRUB; *Clostridium pasteurianum* (P23171), CT_YRUB; *Clostridium thermocellum* (YP_001036667), TP_YRUB; *Thermotoga petrophila* (YP_001245300), FN_YRUB*;* *Fervidobacterium nodosum* (YP_001410364), TM_YRUB; *Thermosipho melanesiensis* (YP_001305804), PM_YRUB; *Petrotoga mobilis* (YP_001568453), BSP_YRUB; *Bacillus* sp. (ZP_01860678), BW_YRUB; *Bacillus weihenstephanensis* (YP_001646612), BC3987; *Bacillus cereus* (NP_833706), BAA_NRDH; *Bacillus anthracis* (YP_030147), BC_YRUB; *Bacillus cereus* subsp. *Cytotoxis* (YP_001375918), SA_YRUB; *Staphylococcus aureus* (YP_185957).
